# Physical and Functional Properties of Toothpaste Tablets

**DOI:** 10.3390/ma18204804

**Published:** 2025-10-21

**Authors:** Agata Blicharz-Kania, Justyna Kot, Dariusz Andrejko

**Affiliations:** Department of Biological Bases of Food and Feed Technologies, University of Life Sciences in Lublin, Głeboka 28, 20-612 Lublin, Poland

**Keywords:** toothpaste tablets, fluoride, waterless cosmetic, friability, strength, functional properties

## Abstract

Products such as toothpaste tablets align with the concept of sustainable cosmetic production. The aim of this study was to evaluate the physical and functional properties of toothpaste tablets with different formulations—with and without fluoride, surfactants, and dried herbs. The following parameters were determined: friability (using a shaking method), compressive strength (using a tensile testing machine), colour parameters (spectrophotometrically), pH, and foaming capacity. The study results showed that tablet durability is closely dependent on the formulation. Tablets made with commonly used ingredients (control sample) had the highest breaking force (55.24 N). Tablets without fluoride had the lowest friability (1.46%). Optical tests showed that different formulations affected tablet brightness and colour saturation. The largest changes were observed for samples containing dried herbs—Δ*E* > 5. The tablets with clove added had improved foam quality, which is important from a functional perspective. The disintegration time of the tablets was significantly shorter for the modified formulation samples. The study results indicate that the developed tablets, especially the control and fluoride-free samples, are sufficiently hard and durable. The tablets with added herbal ingredients, on the other hand, exhibit good foaming and dissolving properties and are waterless products without preservatives.

## 1. Introduction

Oral health significantly impacts overall health and quality of life. The health of oral tissues determines basic life functions such as eating, speaking, and social interactions, which also impact a person’s mental health [1]. Oral and gum care is a key aspect of personal healthcare. Various types of cosmetics and oral hygiene devices are available on the market. These include toothbrushes, miswaks, irrigators, dental floss, toothpastes, mouthwashes, oral sprays, powders, and tablets [2]. In cosmetics containing water, preservatives play a special role, as they must meet numerous legal and toxicological requirements. They are selected based on criteria such as solubility, stability across various pH and temperature ranges, lack of interaction with other cosmetic ingredients, and resistance to light and oxygen. Annex V of Regulation (EC) No. 1223/2009 of the European Parliament and of the Council of 30 November 2009 [3] on cosmetic products outlines the preservatives permitted in cosmetic products. Due to the occurrence of undesirable side effects, such as allergic reactions or skin irritations, cosmetics manufacturers are increasingly seeking natural preservatives and solutions that reduce the use of preservatives. Martins and Mart [4] mention oral hygiene cosmetics, emphasizing that there are natural plant-based preservatives that can be used in such products. Citrus extracts are one example used in oral hygiene products. Plant materials may also be effective in treating dental caries. Lemon oil exhibits antibacterial and antimicrobial activity, and has been shown to have a beneficial effect in inhibiting dental biofilm formation and may be used as an alternative adjunct to anticaries therapy [5]. Toothpaste containing lemon oil has been shown to be effective as a remineralizing agent [6]. Peppermint oil is widely used in oral hygiene products due to its antibacterial and refreshing properties. Peppermint oil may exhibit antiseptic activity against oral pathogens [7]. Good antibacterial and antibiofilm activity has been confirmed even at low concentrations [8]. Chamomile, in turn, has beneficial anti-inflammatory and antiseptic effects, and the effectiveness of chamomile gel in treating oral diseases has been confirmed in clinical trials [9]. Carl & Emrich [10] demonstrated that chamomile mouthwashes alleviated inflammation of the oral mucosa caused by radiotherapy. Other natural raw materials with strong antibacterial properties are cloves and the eugenol they contain [11]. Clove essential oil has antimicrobial activity against oral bacteria [12].

The development of tablet-based products has also been described; they are more environmentally friendly because they reduce water consumption and packaging waste. Cosmetic manufacturers are proposing preservative-reduced waterless cosmetics. These are formulations that are water-free or contain only trace amounts of water. Eliminating water from formulations offers numerous advantages, such as reducing the amount of preservatives and other excipients, increasing the proportion of natural substances, and obtaining a highly concentrated product rich in active ingredients. Faced with the global water crisis, the cosmetics industry is taking steps to reduce water consumption [13]. Toothbrush tablets are an innovative solution that eliminates the need for traditional toothpaste tubes, which are often not fully recycled. The tablets are easy to use—just chew them and brush your teeth as usual. They are packaged in reusable packaging or made from recyclable materials, which reduces waste. Additionally, such products often contain no artificial colours or preservatives, making them more health-friendly [14,15]. Positive consumer responses to toothpaste tablets have been demonstrated, confirming their potential as a sustainable alternative to traditional toothpastes [16,17].

Selecting the appropriate manufacturing method for toothpaste tablets and developing the optimal composition of these products can contribute to the creation of an ecological and economical alternative to traditional toothpaste [18]. Various studies have been conducted on the life cycle of toothpaste creams and tablets, the effectiveness of using tablets instead of pastes, the impact of toothpaste formulation on plaque removal effectiveness, and sensory acceptability by users [15,17,19,20,21]. However, little attention has been paid to the physical or functional properties of toothpaste tablets. These properties are crucial for both transport and use of the product. Tablets with poor durability may crumble during transport and other handling activities. The user will receive an inferior product. Furthermore, hardness must not be too high or too low. Tablets with low hardness may be damaged, for example, when removing them from the packaging or picking them up. However, too high a hardness can make them difficult to crack/chew. Tablets must therefore be resistant to abrasion and cracking and possess a certain mechanical strength after formation. Other important tablet properties that must also be controlled include disintegration/dissolution time and foaming capacity. Formulation and process factors will likely influence the final quality of these types of products. The article presents a research hypothesis according to which modifying the recipe of toothbrush tablets will significantly affect their physical and functional properties. This study analysed the effect of tablet composition, including the addition of dried herbs, on selected properties of toothpaste tablets. Analysis was conducted on the friability, compressive strength, colour parameters, acidity, foaming capacity, and disintegration time of the tablets.

## 2. Materials and Methods

### 2.1. Research Material

The research material consisted of toothbrush tablets made from the following ingredients: xylitol, calcium carbonate, white clay, microcrystalline cellulose, sodium bicarbonate, magnesium stearate, SCI (Sodium Cocoyl Isethionate), sodium fluoride, lemon oil, peppermint oil, dried chamomile, and dried cloves. Five powder mixtures were prepared, as shown in Table 1.

### 2.2. Preparation of Tablets

Xylitol, chamomile, and cloves were ground in a laboratory mill (Chemland, FW100, Anseong-si, Republic of Korea). The powders were sifted through a 0.5 mm mesh sieve and weighed in the appropriate quantities according to Table 1. They were premixed in a mortar and pestle, then thoroughly combined in a crusher (Tefal HB 655, Rumilly, France). The resulting mixtures were tableted using 9 mm diameter punches.

**Figure 1:**
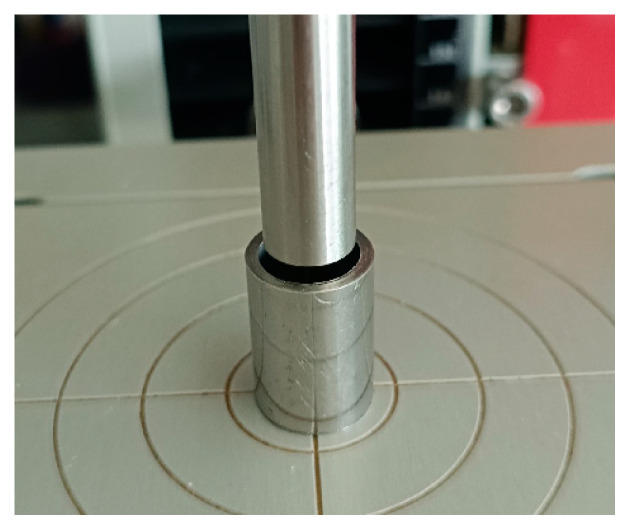
Punches for tablet preparation.

The resulting mixtures were tableted using a compression machine (Zwick/Roell BT1-FR0.5TN.D14, Ulm, Germany) (Figure 1). The plunger force was set to 500 N. The process was carried out in three compression cycles. The established methodology was based on previously conducted preliminary research. The compression speed was 0.25 mm/s. The head, pressing on the plunger, allowed the formation of a single tablet weighing approximately 250 mg and measuring 9 mm in diameter.

### 2.3. Tablet Testing

The resulting tablets were tested for durability and strength (crush resistance), and their time, pH, foaming capacity, and mineral content were measured.

#### 2.3.1. Uniformity of Weight and Thickness Test

The thickness, height, and mass of 5 randomly selected tablets were measured using a caliper with an electronic display (Limit, Alingsås, Sweden) and a RADWAG AS 310.R2 laboratory scale (Radom, Poland).

#### 2.3.2. Friability Test

Friability is a measure of the tablets’ resistance to shocks resulting from transportation, various processes related to distribution, and use. To determine durability, a shaking test was performed in which the tablets collided with each other and with the walls of the container. Friability was calculated as the ratio of the sample mass loss remaining after the test to the initial sample mass (*m*_1_), according to Formula (1). Measurements were performed using a laboratory shaker (ELMI Orbital Shaker S-3.02.20M, Milano, Italy) at a rotational speed of 200 rpm; the test time was 5 min, and the initial sample mass was approximately 1.25 g.

(1) $$Friability=\frac{m_{1}-m_{2}}{m_{1}}\;\left(\%\right)$$

where:

*m*_1_—initial tablet sample mass [g],
*m*_2_—tablet sample mass after shaking [g] [22].

#### 2.3.3. Strength (Crush Resistance)

The test involved placing the sample horizontally between the working plates of the measuring head of the previously mentioned Zwick/Roell device. The product was crushed with a 5 mm diameter cylindrical piston, measuring the pressure at which the tablet crushed [22]. The test continued until the sample fractured. The test was repeated five times for all tablet types. Based on the parameters recorded by the device, a force-deformation (*F-s*) curve was determined. The tablet strength (breaking force) was measured as the maximum force (*F_max_*) recorded during the test.

#### 2.3.4. Colour Parameter Measurement

A 3Color^®^ SF80 spectrophotometer (Narama, Poland) was used for the analysis. Five replicates were performed for each test sample (D65 light source, 10° observer, 8 mm measuring head aperture) [23]. The following parameters were measured: L*—describes brightness, a*—describes colour changes from green to red, and b*—describes colour changes from blue to yellow (higher a* and b* values indicate greater red and yellow intensity, respectively).

The total colour change Δ*E* was calculated using the Formula (2):

(2) $$∆E=\sqrt{{∆L}^{2}+{∆a}^{2}+{∆b}^{2}}$$

#### 2.3.5. pH Testing

1 g of each preparation was dispersed in 10 mL of purified water (pH 6.98), and the pH was measured in triplicate using a digital pH meter (pH 780 Metrohm, Herisau, Switzerland) [24].

#### 2.3.6. Foaming Capacity Determination

The foaming capacity of the toothpaste tablets was assessed by grinding and mixing 5 g of the preparation with distilled water (50 mL). The mixture was transferred to a graduated cylinder. The initial volume *V*_1_ was recorded and then shaken 10 times. The final volume of foam + water *V*_2_ was recorded. Foaming capacity was calculated as *V*_2_ − *V*_1_. The experiments were performed in triplicate with 5 g of each preparation [24]. The foam volume formed in the cylinder was measured again 10 min later. Based on these measurements, the foaming stability was determined.

#### 2.3.7. Determination of Disintegration Time

Three randomly selected tablets of each type were analysed. One tablet was placed in a beaker containing 100 mL of distilled water at 37 °C [22], then the vessel was placed in a water bath (AJL electronic MLL 547, Cracow, Poland). The tablets were observed for dissolution, and their disintegration time was recorded.

#### 2.3.8. Statistical Analyses

The obtained results were statistically analysed using Statistica 13 software. One-way ANOVA was performed. Significance of differences was verified using Tukey’s test, at a significance level of α = 0.05. Results are presented as mean values ± standard deviation.

## 3. Results and Discussion

**Figure 2:**
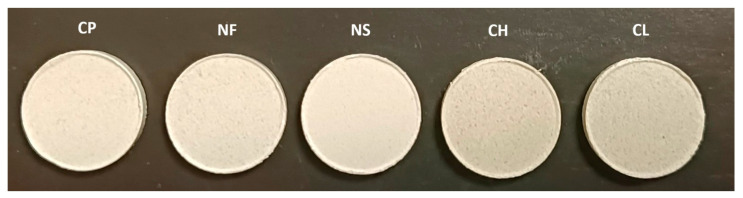
Produced toothpaste in tablet form. CP—control probe (toothpaste in tablet form), NF—fluoride-free tablets, NS—SCI-free tablets, CH—tablets with dried chamomile, CL—tablets with dried cloves.

Tablets produced by direct compression are shown in Figure 2. They differed in colour and thickness. Analysis of these parameters is presented later in this paper.

Based on the results of the statistical analysis, it was noted that the basis weight (weight and thickness) of the CP and NF samples did not differ significantly. No significant differences in these parameters were observed between the CH and CL samples. The numerical data presented in Table 2 indicate that tablets with an average weight ranging from 229.8 mg (NS) to 244.8 mg (NF) were obtained. Removing fluoride from the tablet formulation had a significant impact on their weight. Other formulation modifications resulted in a reduction in the average tablet weight. The largest changes were observed for the NS sample. These changes indicate increased powder losses during production. The material likely adhered more to the machine walls. Small changes in the average tablet weight may occur, for example, after using different excipients [25]. Sinka et al. [26] emphasize that the same powder formulation can have different performance on different presses. The thickness of the obtained toothpaste in tablet form generally did not differ significantly for most formulations. The exception was the sample without SCI. The NS tablets were significantly thicker—by 0.05 mm—than the control sample. Changes of this magnitude are a normal phenomenon during direct compression tableting of mixtures with slightly different compositions [22,25,27]. Therefore, the test results will be primarily relevant to the manufacturer. Once the mixtures are put into production, it will be necessary to determine the appropriate powder input weight. However, it should be remembered that the change in tablet weight will also have clinical significance—a smaller formulation typically contains fewer active ingredients and may be slightly less effective.

After compression, the manufactured toothpaste tablet undergoes various processes, such as bulk handling, during which it must maintain its mechanical integrity. Tablets must also be capable of disintegrating or dissolving in the oral cavity to achieve the required bioavailability after administration. These properties depend on the composition and the selection of manufacturing process parameters [26]. The crush resistance of tablets is important for both the manufacturer and the user. The high strength of these products ensures tablet stability during transport. From the user’s perspective, the crush resistance should be high enough to prevent the tablet from disintegrating in the hand, but not too high to allow for crushing and chewing.

In the presented results, friability ranged from 1.46% to 5.30% depending on the tablet formulation. Significant differences in the parameter were demonstrated for each of the samples. Fluoride-free tablets demonstrated the highest durability. The effectiveness of fluoride has been confirmed in numerous clinical studies, which have shown that various forms of fluoride (such as toothpastes, varnishes, mouthwashes, and supplements) significantly reduce the incidence of caries, especially in high-risk groups such as children, orthodontic patients, and the elderly [28,29]. Fluoride’s mechanism of action involves inhibiting enamel demineralization, promoting remineralization of early carious lesions, and inhibiting the activity of cariogenic bacteria in dental plaque [30]. Furthermore, fluoride affects bacterial metabolism, limiting their ability to produce acids from carbohydrates, further reducing the risk of caries [31].

Roberts and Rowe [32] analysed the compression properties and mechanical strength of tablets, focusing on excipients such as microcrystalline cellulose. Their studies showed that excipients that promote uniform distribution of compression forces increase tablet strength. In the study by Haruna et al. [25], where tablets were made from microcrystalline cellulose, an average tablet friability of 0.61% was achieved—0.82% less compared to other formulations. These results indicate that tablets with microcrystalline cellulose exhibited significantly lower crumbling losses compared to other excipients.

The addition of dried herbs in our study simultaneously reduced the microcrystalline cellulose content. It can be assumed that the addition of a larger amount of this raw material would have provided adequate crumbling strength. From a clinical application perspective, adequate tablet hardness and mechanical integrity are essential for ensuring product stability during transport, storage, and daily patient use. Tablets with insufficient hardness may crumble prematurely, resulting in uneven release of active ingredients and potentially compromising the effectiveness of their anticariogenic and antibacterial effects [33,34]. Microcrystalline cellulose is a commonly used filler and binder in tablet formulations, known for its beneficial compression properties and ability to maintain the tablet’s structural integrity [35]. Its reduction in the tested samples due to the use of dried herbs may have contributed to a decrease in mechanical strength. Therefore, increasing the cellulose content or using alternative excipients may be important in further improving the formulation to maintain both the required physicochemical parameters and clinical efficacy.

Toothpaste tablets must have sufficiently low friability and high hardness to prevent damage during transport. However, they must not be too hard to allow chewing before brushing. By far the highest breaking force was recorded for the control sample. All formulation modifications resulted in a significant reduction in compressive strength. The lowest breaking force values were observed for the NS and CL samples. On the one hand, the introduction of a powdered surfactant allowed for a significantly more durable tablet. However, the addition of dried herbs, especially cloves, significantly reduced this durability. In addition to the formulation ingredients, the tablet manufacturing method is also an important factor. Studies conducted by Mohylyuk et al. [36] on chewable tablets have shown that lower compression forces promote better textural profiles and release of active ingredients, although they can lead to surface imperfections and particle alignment. The authors observed a proportional relationship between tablet hardness and compression force. The higher the compression force, the greater the tablet hardness. However, high pressure (>10 kN) increased the percentage of tablets with defects after the friability test. Another study analysed the effect of compression force on various properties of chewable tablets containing drugs. Higher compression forces resulted in harder and more adhesive gums. However, this can lead to chewing difficulties and reduced patient acceptability. The optimal compression force for these tablets was 7 kN, which provided a good balance between hardness and adhesion while meeting pharmacopoeial requirements [37]. Compression force directly affects tablet hardness, which in turn can affect tablet texture (e.g., friability or breakability), disintegration time, and the active ingredient release profile. Excessive compression force can result in tablets that are too hard, which can make disintegration difficult and reduce the active ingredient’s bioavailability. For patients, the right tablet texture and hardness not only impact comfort but also therapeutic compliance. Tablets that disintegrate too slowly and are difficult to chew may cause irritation, damage to teeth, and may be reluctant to be used by the user [38].

The results of the statistical analysis indicate that the formulation significantly affects the optical properties of toothpaste tablets (Table 3). Tablets from formulas CP and NS have the highest brightness and are statistically comparable (no significant differences). Formulations G and R have lower brightness and are significantly darker than the others. Tablets from formula CL are characterized by the highest red colour saturation. The remaining formulations had significantly lower, similar a* values. Samples CP, NS, and NF did not differ significantly in terms of the a* parameter. Tablets from formula CH are characterized by the highest yellow colour saturation. For tablets manufactured according to the NS and CL recipes, no statistically significant differences in the b* parameter were found. The control sample had the lowest yellowness. Similar correlations were demonstrated in studies conducted by Hodžić et al. [39] which found that changes in formulation can significantly affect the mechanical and optical properties of tablets. In their study, tablets with a high concentration of *Hypromellose* had a brightness (L*) of 85.32, a red colour saturation (a*) of 3.45, and a yellow colour saturation (b*) of 8.67. Tablets with added cellulose microcrystals had a brightness of 78.95, a red colour saturation of 2.80, and a yellow colour saturation of 7.32. In turn, tablets with a lower amount of *Hypromellose* and a higher rolling pressure had a brightness of 70.45, a red colour saturation of 4.10, and a yellow colour saturation of 6.15.

The total colour change was compared to the control sample. The smallest differences were noted for the NS sample (Δ*E* < 1—the observer sees no difference). The total colour change for the fluoride-free tablets was so small that only experienced observers could notice the difference. However, attention should be paid to significant changes in colour parameters for tablets with added chamomile or cloves Δ*E* > 5 (observers notice two different colours) [40]. These changes are related to the different colour of these raw materials compared to the colour of the other ingredients used. In a study by Catani et al. [41], it was indicated that chamomile contains chemical compounds such as flavonoids and volatile oils, which may be responsible for its colour. Infusions and essential oils from fresh or dried flowers have aromatic, flavouring and colouring properties. The green-blue colour of chamomile oil comes from azulene [42]. It has been shown that chamomile extracts can be successfully used to dye cotton fabrics [43].

Changes in the optical properties of tablets (e.g., colour, gloss) can have significant clinical implications. They can affect patient recognition and acceptance of the drug, which is crucial for adherence to therapy—patients often identify medications visually. Uncontrolled changes can lead to errors or reduced confidence in the product’s quality [44]. Furthermore, optical changes can signal degradation processes, which can be interpreted as a change in the bioavailability of the active ingredient [45].

For enamel health, a neutral or alkaline pH is best, which prevents demineralization. The pH of all samples studied fell within this range (Table 4). Statistical analysis results suggest that different toothpaste formulations affect their pH. The control sample has a pH comparable to CH. However, NS tablets exhibit a significantly higher pH, indicating that the presence of SCI significantly reduces the tablet pH. The surfactant used tends to lower the pH of solutions, which may affect the stability and effectiveness of various cosmetic and pharmaceutical formulations. Studies on gels for application to the skin have shown that SCI can lower the pH of gels, which is beneficial for maintaining the skin’s protective acid mantle, which protects against pathogens and maintains the proper skin microflora [46]. Regarding the pH of oral hygiene products, a neutral or slightly alkaline pH is recommended. Toothpaste pH can generally range from 7 to 10, depending on its additives. Furthermore, toothpaste pH also has implications for consumers’ oral health. Toothpaste is not manufactured at a low pH because it could dissolve enamel and dentin minerals [47].

Toothpaste pH plays a key role not only in its effectiveness but also in maintaining oral health. Fernandez et al. [48] demonstrated that appropriately adjusted pH promotes enamel remineralization and limits demineralization. This is crucial for preventing dental caries and erosion. It is the pH value that determines whether the environment favours enamel reconstruction or contributes to its degradation [48]. This relationship is also confirmed by the results of a study by Mehrjoo et al. [49], which assessed the effect of a toothpaste containing nano-hydroxyapatite on enamel erosion induced by orange juice. The authors demonstrated that using a toothpaste with a pH close to neutral significantly reduced the loss of enamel hardness and supported its remineralization. Importantly, the effectiveness of this toothpaste was linked not only to the presence of active nanoparticles but also to maintaining an appropriate pH, which prevented further acidification of the oral environment and promoted repair processes. According to Farooq et al. [50], the effectiveness of toothpastes in the remineralization process depends not only on the presence of fluoride but also on the chemical composition of the environment, particularly the pH value. A properly balanced pH promotes the absorption of calcium and phosphate ions by the enamel, supporting its reconstruction and strengthening the natural protective barrier against acid. However, too low a pH can irritate the oral mucosa and intensify demineralization processes. Conversely, too high a pH can limit the effectiveness of some active ingredients contained in toothpaste.

A different relationship can be observed for samples without fluoride. The presence of this ingredient may shift the pH toward a more alkaline state. The findings of the study by Setiawan et al. [51] indicate a significant difference in salivary pH before and after brushing with fluoride toothpaste; a significant difference in salivary pH before and after brushing without toothpaste; and a significant difference in salivary pH before and after brushing with and without fluoride toothpaste. However, it is worth noting the changes induced by the addition of dried cloves. Herbs may contain organic acids (salicylic, valeric, isochlorogenic) [52] and phenols (eugenol, gallic acid) [53], therefore, when using them, the physicochemical changes that may occur should be considered. The cloves used in the study lowered the pH of the tablets. The main component of cloves is eugenol (a phenolic aromatic compound). Eugenol and other phenols are slightly acidic. Cloves also contain organic acids (including gallic acid, ellagic acid, and oleanolic acid). Cai and Wu [54] demonstrated that clove extracts can inhibit the growth of cariogenic bacteria but can also cause a temporary decrease in the pH of saliva or the solution in which they are present. This phenomenon may be partially beneficial—bacteria multiply more slowly in an acidic environment—but in excess, too low a pH promotes enamel demineralization.

The results of the statistical analysis suggest that the addition of chamomile to the basic tablet formulation did not significantly affect foaming, nor did the exclusion of fluoride from the formulation (Table 4). However, statistically significant differences in FA were observed between samples NF, NS, CH, and CL. Tablets with the addition of chamomile or cloves exhibited the highest foaming capacity. However, the foaming capacity of CH did not differ significantly from the control sample. However, the foaming capacity of the remaining tablet samples was significantly lower, with the smallest changes observed for the NF sample. Tablets without SCI exhibited the lowest foaming capacity, which can be explained by the typical properties of surfactants. Similar results were obtained by Gupta et al. [55], who analysed the effect of various foaming agents on foaming and foam stability. They showed that the addition of SCI significantly increased foam volume from 200 mL to 350 mL and improved foam stability from 10 min to 25 min. Fichtner and Schubert [56] analysed the effect of SCI on the mechanical properties of tablets and found that SCI improved tablet foaming by better distributing surfactants within the tablet matrix. Hadjiiski et al. [57] analysed the effect of various oil additives on the foaming and foam stability of various surfactants, including SCI. They found that SCI created a less stable foam compared to sulphate surfactants. Interestingly, tablets containing dried cloves showed the highest foaming properties. In addition to the phytochemicals mentioned above, clove buds also contain saponins—glycosides that lower the surface tension of water, leading to foam formation [58]. This is important from a user’s perspective, as foam quality influences the sensation of brushing and the effectiveness of stain removal.

The results of the statistical analysis suggest no significant differences in foam stability between the CP and other samples. However, significantly greater foam stability was observed for the tablet solution containing dried chamomile or cloves compared to the sample without SCI. Herbal additives may therefore represent a potential alternative to previously used surfactants. The study results show that biosurfactants present in plant extracts can be used as cosmetic raw materials. Their usefulness in creating formulations requiring surface tension reduction and emulsion formation was confirmed [59].

A tablet that dissolves or disintegrates rapidly in the mouth upon contact with saliva creates a solution or suspension of the administered medication [60]. A short disintegration time is important for the active ingredients to be quickly released and effective in the mouth. A tablet that disintegrates quickly in the mouth, without prolonged chewing, will also be more favourably evaluated by the user. The results of the statistical analysis suggest that tablet formulations influence their disintegration time. The control group (K) demonstrated significantly higher solubility compared to the other groups. The disintegration time of the NF tablets was significantly shorter (over 1 min) compared to the CP. The presence of fluoride may therefore hinder tablet disintegration in water. In turn, the use of herbal supplements (chamomile, cloves) reduced the disintegration time of the tablets compared to the control group. Tablets without SCI (NS) showed the shortest disintegration time, suggesting that the presence of this ingredient significantly increased the disintegration rate of the tablets. A rapid disintegration time is essential for rapid release of active ingredients and their effective action in the oral cavity. In the study by Zimmer et al. [61], tablets containing MCC had a disintegration time ranging from 86 to 161 s. In the study by Gozdur et al. [62], the addition of microcrystalline cellulose (Vivapur^®^ 102) prolonged the disintegration time of the tablets. Tablets with microcrystalline cellulose typically had a longer disintegration time than those without it, with some batches exceeding 15 min.

In the study by Marczyński and Bodek [63], a Korsch EK-O impact tablet press with concave punches with a 12 mm diameter was used to produce tablets with *Epilobium parviflorum* herb extract. The disintegration time of the tablets immediately after preparation ranged from 12 to 53 min, depending on the batch. After 12 months of storage, this time increased slightly but still remained within acceptable limits. The results indicate that the disintegration time of the control tablets (using microcrystalline cellulose) was 443 s. In Haruna et al. [25], the disintegration time of the tablet made from microcrystalline cellulose was over 15 min (900 s). Tablets made from microcrystalline cellulose had greater durability and a slower disintegration time, which may be due to their higher mechanical strength and structure, which was more resistant to water. However, the opposite relationship was observed in the NS tablets; they were characterized by high hardness and low friability, while having a very short disintegration time.

The solubility of toothpaste tablets plays a significant role in their clinical effectiveness, particularly in the context of controlling bacterial biofilm and preventing caries. Rapid and uniform tablet dissolution enables the effective release of active ingredients, such as antibacterial agents (e.g., essential oils, chlorhexidine) and remineralizing ingredients (e.g., fluoride, hydroxyapatite), allowing for effective action on tooth surfaces and biofilm [34,64]. Inadequate solubility can limit the contact of active ingredients with areas susceptible to demineralization, reducing their preventive effect. Furthermore, effective dissolution is crucial for enabling the mechanical action of the foam and surfactants, which aid in plaque removal [65].

## 4. Conclusions

This study confirmed that different formulations have a significant impact on the physical and functional properties of toothpaste tablets. The results indicate the potential for developing natural tablets with high durability and effectiveness, which could serve as an alternative to traditional oral hygiene products. It was shown that tablet durability is closely dependent on the formulation. Tablets made with commonly used ingredients (control sample) and those without fluoride had the lowest friability and the highest breaking force. The SCI additive was found to significantly improve foam quality, which is crucial for toothbrushing effectiveness. Tablets with dried chamomile also demonstrate high foaming capabilities. Therefore, it is possible to use this raw material as an alternative to traditional surfactants. However, it should be noted that this raw material significantly lowers the pH of toothpaste tablets and adversely affects their friability and hardness. The tested tablets, especially those with a modified formulation, disintegrated at the appropriate time, ensuring their effectiveness in daily use.

Continued research on formulation optimization and analysis of tableting process parameters could lead to further increases in shelf life and reduced crumbling losses. Exploring new, natural additives could improve the mechanical and functional properties of tablets while simultaneously increasing their health-promoting value. Furthermore, further research on the impact of various additives on consumer acceptability could lead to the development of formulations that are both stable and well-received by users.

## Figures and Tables

**Table 1:** Formulation of the obtained toothpaste in tablet form.

Formulation/Ingredients	Ingredient Amount [g]	Type of Tablets				
		CP	NF	NS	CH	CL
Xylitol	15	+	+	+	+	+
Calcium carbonate	10	+	+	+	+	+
White clay	5	+	+	+	+	+
Microcrystalline cellulose	5	+	+	+	+	+
Sodium bicarbonate	5	+	+	+	+	+
Magnesium stearate	4	+	+	+	+	+
SCI (Sodium Cocoyl Isethionate)	4	+	+	−	+	+
Sodium fluoride	1	+	−	+	+	+
Lemon oil	0.5	+	+	+	+	+
Peppermint oil	0.5	+	+	+	+	+
Dried chamomile	1	−	−	−	+	−
Dried cloves	1	−	−	−	−	+

+ presence of an ingredient; − absence of an ingredient; CP—control probe (toothpaste in tablet form), NF—fluoride-free tablets, NS—SCI-free tablets, CH—tablets with dried chamomile, CL—tablets with dried cloves.

**Table 2:** Characteristic of the obtained toothpaste in tablet form.

Probe	Weight Uniformity ^1^ (mg)	Tablet Thickness ^1^ (mm)	Friability ^2^ (%)	Breaking Force ^1^ (N)
CP	244.6 ± 3.2 ^a^	2.96 ± 0.01 ^b^	1.88 ± 0.08 ^d^	55.24 ± 0.42 ^a^
NF	244.8 ± 3.3 ^a^	2.95 ± 0.02 ^b^	1.46 ± 0.01 ^e^	48.46 ± 1.26 ^b^
NS	229.8 ± 1.8 ^c^	3.01 ± 0.01 ^a^	5.30 ± 0.11 ^a^	25.74 ± 1.18 ^e^
CH	235.8 ± 2.5 ^b^	2.99 ± 0.03 ^ab^	2.54 ± 0.12 ^c^	45.78 ± 1.83 ^c^
CL	234.0 ± 2.3 ^bc^	2.96 ± 0.01 ^b^	3.39 ± 0.22 ^b^	38.02 ± 1.25 ^d^

CP—control probe (toothpaste in tablet form), NF—fluoride-free tablets, NS—SCI-free tablets, CH—tablets with dried chamomile, CL—tablets with dried cloves. The presented numbers represent the arithmetic mean of the measurements (^1^ *n* = 5, ^2^ *n* = 3) ± SD. Data value of each parameter with different superscript letter in column are significantly different (Tukey test results: Weight Uniformity: *p* ≤ 0.0001, Tablet Thickness: *p* = 0.0004, Friability: *p* ≤ 0.0001, Breaking force: *p* ≤ 0.0001).

**Table 3:** Colour parameters of the obtained toothpaste in tablet form.

Probe	L*	a*	b*	Δ*E*
CP	88.84 ± 0.84 ^a^	0.25 ± 0.06 ^b^	4.72 ± 0.02 ^d^	-
NF	87.05 ± 0.56 ^b^	0.26 ± 0.05 ^b^	4.97 ± 0.06 ^c^	1.80
NS	88.80 ± 0.41 ^a^	0.27 ± 0.03 ^b^	5.19 ± 0.14 ^bc^	0.47
CH	83.47 ± 0.93 ^c^	0.13 ± 0.02 ^c^	7.52 ± 0.19 ^a^	6.06
CL	83.67 ± 1.15 ^c^	0.96 ± 0.08 ^a^	5.38 ± 0.11 ^b^	5.26

CP—control probe (toothpaste in tablet form), NF—fluoride-free tablets, NS—SCI-free tablets, CH—tablets with dried chamomile, CL—tablets with dried cloves. The presented numbers represent the arithmetic mean of the measurements (*n* = 5) ± SD. Data value of each parameter with different superscript letter in column are significantly different (Tukey test results: L*: *p* ≤ 0.0001, a*: *p* ≤ 0.0001, b*: *p* ≤ 0.0001).

**Table 4:** The functional properties of the obtained toothpaste in tablet form.

Probe	pH (-)	FA (cm^3^)	FS (cm^3^)	Disintegration Time (min:s)
CP	7.87 ± 0.02 ^b^	15.67 ± 0.58 ^bc^	0.93 ± 0.12 ^ab^	7:23 ± 0:15 ^a^
NF	7.77 ± 0.04 ^cd^	13.67 ± 0.58 ^c^	0.930 ± 0.12 ^ab^	6:08 ± 0:21 ^b^
NS	8.19 ± 0.04 ^a^	0.83 ± 0.29 ^d^	0.40 ± 0.17 ^b^	4:04 ± 0:11 ^d^
CH	7.83 ± 0.03 ^bc^	17.57 ± 1.25 ^b^	1.16 ± 0.29 ^a^	4:44 ± 0:06 ^c^
CL	7.71 ± 0.03 ^d^	22.07 ± 1.40 ^a^	1.13 ± 0.40 ^a^	5:12 ± 0:13 ^c^

CP—control probe (toothpaste in tablet form), NF—fluoride-free tablets, NS—SCI-free tablets, CH—tablets with dried chamomile, CL—tablets with dried cloves. The presented numbers represent the arithmetic mean of the measurements (*n* = 3) ± SD. Data value of each parameter with different superscript letter in column are significantly different (Tukey test results: pH: *p* ≤ 0.0001, FA: *p* ≤ 0.0001, FS: *p* = 0.0221, Disintegration time: *p* ≤ 0.0001).

## Data Availability

The original contributions presented in this study are included in the article. Further inquiries can be directed to the corresponding author.
